# From exception to insight: a phenomenological study of quality managers’ perspectives on the individual appeals in the National Healthcare Quality Program

**DOI:** 10.1186/s13584-026-00766-z

**Published:** 2026-06-15

**Authors:** Khaled Awawdi, Elena Maoz, Limor Friedensohn, Yifat Erlich-Shoham

**Affiliations:** 1 Ramat Gan Academic College, 87 Pinhas Rotenberg Street, 5227500 Ramat Gan, Israel; 2 Ministry of Health, Jerusalem, Israel

**Keywords:** Quality Management, Hospital Performance, Individual Appeals, Professional Recognition, Healthcare Quality Indicators, Qualitative Study

## Abstract

**Background:**

National quality indicators play a crucial role in monitoring hospital performance and fostering accountability. However, standardized indicators often fail to capture the intricate realities of clinical practice. To mitigate this limitation, Israel’s National Program for Quality Indicators for general, geriatric, and psychiatric hospitals has implemented an individual appeals process, enabling hospitals to request case-based exclusions from indicator calculations when performance deviations arise from factors beyond their control. After three years of this initiative, our goal is to investigate the experiences of hospital Quality Managers (QMs) with the individual appeals track, aiming to inform enhancements to the national quality indicators program.

**Methods:**

A qualitative, phenomenological study was conducted using semi-structured interviews with ten QMs from general, geriatric, and psychiatric hospitals. Interviews were analyzed using thematic, textual, and content analysis.

**Results:**

Three core themes emerged: (1) Knowledge and familiarity gaps - most QMs relied on experiential learning rather than formal training or Ministry guidelines; (2) Communication gaps - strong collaboration existed within hospitals and with supervising nurses, but limited dialogue and feedback occurred with higher Ministry levels or across institutions; (3) Recognition gaps - QMs perceived acceptance of appeals as professional validation, while rejections often evoked frustration and a sense of underappreciating.

**Conclusions:**

QMs’ experiences highlight systemic gaps in training, communication, and recognition that influence the perceived legitimacy and effectiveness of the individual appeals track. Strengthening structured onboarding, establishing transparent two-way communication, and embedding formal recognition mechanisms could enhance motivation, professional confidence, and the program’s credibility.

**Policy implications:**

Institutionalizing the appeals process as a formal policy tool supported by standardized training, inter-hospital learning forums, and routine feedback loops can transform it from a technical exception mechanism into a strategic platform for continuous quality improvement and policy learning within Israel’s healthcare system.

## Introduction

Quality measurement has become a cornerstone of modern healthcare systems worldwide, [1–4] providing structured frameworks for measuring, monitoring, and enhancing the safety and effectiveness of care [5–7]. By establishing standardized benchmarks, it enables meaningful comparisons across institutions, reveals variations in performance, [8], [9] and reinforces accountability at both the clinical and organizational levels [10].

Despite their value, indicator outcomes do not always fully reflect the quality of care provided. [11, 12] Optimal treatment may not be feasible in certain cases due to circumstances beyond the institution’s control, for example, when a patient declines a recommended procedure, presents too late for timely intervention, or has complex comorbidities that limit the applicability of standard treatment protocols. These scenarios highlight the importance of interpreting quality measures within their clinical and organizational context and of considering individual cases on their own merits.

Therefore, standardized indicators, if applied rigidly, may misclassify clinically appropriate care as poor performance or disadvantage providers who care for more complex or high-risk patients [13]. In addition, a large body of literature has documented that quality measurement can generate unintended consequences and distortions in clinical practice. These include focusing narrowly on measured indicators at the expense of other aspects of care, avoiding complex or high-risk patients who may adversely affect performance metrics, and adapting documentation or treatment decisions to meet targets rather than clinical needs. Such dynamics may undermine patient-centered decision-making and create incentives that conflict with appropriate clinical judgment. [14–16].

To address these challenges, several health systems incorporate case-level exceptions to ensure that performance metrics more accurately reflect clinical judgment and real-world practice. A prominent example is the United Kingdom’s Quality and Outcomes Framework, where physicians can use “exception reporting” to exclude patients from indicator calculations due to patient refusal, clinical contraindications, or other legitimate circumstances [17]. Similarly, some programs in the United States allow case-level exclusions when clinical contraindications or documented patient preferences justify deviation from guideline-based care. [18, 19]. Systematic reviews and evaluations show that exception reporting mitigates the unintended consequences of rigid performance measurement, influences clinician behavior, and maintains the credibility of quality programs. [20–22].

To address this issue, the Israeli National Program for Quality Indicators for general, geriatric, and psychiatric hospitals introduced an “individual appeals track” in 2021. This process is designed to ensure methodological transparency, standardization, and fairness in the evaluation of hospital performance. Comprehensive guidelines [23, 24] for submitting individual appeals were published and made accessible to all Quality Managers (QMs) in hospitals reporting to the National Quality Program. These guidelines included not only procedural instructions but also illustrative examples of specific cases within selected indicators, clarifying the types of circumstances that may warrant an appeal and ensuring transparency and consistency in the process.

According to the guidelines, each QM may request the exclusion of specific patient cases from quality indicator calculations when noncompliance with performance targets results from circumstances beyond the hospital’s control. Each quarter, QMs review reported cases and identify those that merit reconsideration. For each case, they record a justification for exclusion in a designated “Exceptions” sheet within the official reporting file and may consult with a supervising nurse from the Ministry of Health to ensure appropriateness. Once submitted, the Ministry reviews each appeal, examining the clinical and contextual reasoning before issuing an official decision. If approved, the case is removed from the indicator dataset, ensuring that performance evaluations reflect the true quality of care rather than exceptional or uncontrollable events. This structured process promotes transparency, fairness, and context-sensitive quality assessment across hospitals.

Three years after the introduction of the individual appeals track and the publication of comprehensive guidelines, it became essential to explore the experiences of hospital QMs with this process. This qualitative assessment aimed to inform future refinements and ensure that the program continues to support fair, transparent, and context-sensitive evaluation of hospital clinical performance.

## Methods

### Study design

A qualitative, interview-based study was conducted among QMs from general, geriatric, and psychiatric hospitals, with a focus on their experiences and perceptions of the individual appeals track.

A phenomenological approach was utilized, employing semi-structured, in-depth interviews designed to encourage participants to share their experiences and express their thoughts and emotions in a broad and reflective manner [25, 26]. The objective was to examine the participants’ lived experiences in order to reveal the underlying meanings and shared essence of the phenomenon, as understood from their subjective perspectives. Data collection continued until theoretical saturation was achieved [27].

### Study sample

To ensure a comprehensive understanding of the topic, participants were recruited from ten hospitals varying in size and geographic location, and area of specialization:. six QMs were from general hospitals with mixed departments, including geriatric and/or psychiatric units. Two QMs were from exclusively geriatric hospitals, and two from exclusively psychiatric hospitals. Inclusion criteria for the QMs required at least one year of experience in their current role.

All but one of the interviewees were registered nurses holding at least a bachelor’s degree. Among them, nine QMs had obtained a master’s degree, and five had additionally completed an advanced certification course in quality assurance. Regarding cultural background, seven participants were born in Israel and three had immigrated from the former Soviet Union. In terms of self-identified ethnicity, two participants identified as Arab and eight as Jewish. The professional experience range was 13–35 years among the sample, with 1–14 years of the experience in the current role of QM.

### Procedure

An invitation email describing the study was sent to all QMs nationwide, inviting them to participate. Those who consented via email were contacted to schedule a convenient time for the interview. All interviews were conducted by two researchers [KA and EM].

One-time interviews were conducted via Zoom during 2024, each lasting between 30 and 50 min. The interviews began with general questions (e.g., “Describe your education and professional experience”), followed by more focused inquiries based on the interview guide (see Table 1). Clarification prompts were used when necessary, such as “Please explain more” or “Give an example.”

**Table 1:** Interview Guide for Quality Managers

Question
What is your role, your training, your professional seniority, and how long have you been in the role of quality manager?
A few years ago, the process of individual appeals was introduced into routine work. Please explain what this process is, what it includes, and how it conducted in your institution.
Are there official guidelines for this process? If so, what are they? If not, how is the process usually managed in your institution?
What is the place of individual and professional judgment in this process?
Who are the people involved in the process in your institution?
Please describe how the decision to submit an individual appeal is made in your institution.
What are the common reasons for submitting an individual appeal in your institution?
What are the ambiguous or “gray area” cases in reporting? Please provide an example of such a case related to one of the quality indicators.
Based on the case you described, how were such cases typically handled before the introduction of the individual appeal process?
Based on the case you described, how are such cases handled today? Please describe the entire process step by step, including deliberations and decision-making.
When I say “this is a case for an individual appeal” – what comes to your mind? That is, what do you expect?
What do you know about the path an individual appeal takes after it is submitted and before you receive the final decision? Please describe the process.
What is the contribution of the individual appeal process to you as a professional?
What is the contribution of the individual appeal process to the healthcare system?
What is the contribution of the individual appeal process to the quality of care?
Speaking personally, what is your biggest challenge regarding the submission of individual appeals?
What do you know about how this process is conducted in other institutions?
What do you know about this process from the experience of other countries?
If you had unlimited resources and full decision-making authority, what would you change/add/remove from the process?

After each interview, the interviewers held a debriefing session to share impressions and recorded notes excluding identifying information. The process continued until data saturation was reached.

### Data analysis

All interviews were audio-recorded, anonymized, transcribed by the researchers [KA and EM], and analyzed [EM] using thematic, textual, and content analysis techniques [28]. The analysis included repeated, in-depth readings of the transcripts, leading to the development of initial codes and subcodes.

To enhance the credibility and validity of the findings, an additional layer of automated analysis was conducted [KA] using Transcriptor AI, [29] an artificial intelligence based tool for transcription and text analysis. The tool enabled keyword extraction, thematic clustering, and sentiment analysis.

Cross-comparison [KA and EM] between manual and AI-assisted analyses contributed to methodological triangulation and reinforced the trustworthiness of the qualitative interpretations. Any discrepancies between the manual analysis and AI-generated outputs were discussed collaboratively by the researchers, and final coding and thematic decisions were made by consensus. Manual thematic analysis formed the primary basis for determining the final themes, while AI outputs provided additional verification, enriched interpretation, and supported the identification of subtle patterns. This triangulated approach was designed to enhance the credibility and analytical rigor of the study. Specifically, triangulation was achieved through the integration of three complementary sources: (1) direct engagement with the original audio recordings to verify tone, emphasis, and contextual cues not fully captured in text. Additionally, a brief discussion followed each interview to allow for immediate sharing of impressions and preliminary interpretations among the researchers [KA and EM]; (2) thematic analysis conducted by the researcher, allowing for in-depth interpretive insight; (3) automated text analysis to identify key terms, clusters, and sentiment patterns. The convergence of these methods helped reduce interpretive bias and ensured a more robust and nuanced understanding of the qualitative data with the most representative and vivid quotes presented in the findings.

The relative weight of each method was explicitly considered: manual coding and thematic analysis were prioritized in developing the core findings, while AI-assisted outputs were used to triangulate, confirm, and enhance the richness of the themes. This process ensured that the final interpretations reflect both human judgment and data-driven insights, improving rigor and robustness.

### Ethics

The study received approval from the Institutional Review Board of Ramat Gan Academic College (approval number: 1013). All participants signed a written informed consent form, which is kept on file by the researchers, prior to participating in the study. Each participant was interviewed once. Before the interview, the study’s purpose and procedures were explained, and participants’ questions were addressed.

The use of AI-based tools for data analysis was conducted without any identifying information, in accordance with ethical considerations and potential risks outlined in the work of Herdiyanti (2024) [30].

## Results

A qualitative analysis of the interviews identified three main themes: familiarity with the individual appeals track, interactions among stakeholders, and the expectations for professional acknowledge.

### Familiarity with the individual appeals track

A recurring theme across all interviews was participants’ general familiarity with the individual appeals process, which was further divided into four knowledge-related subthemes. The first subtheme concerned engagement with the individual appeals track as part of daily work. The second focused on the sources of knowledge, including onboarding and on-the-job learning. The third addressed familiarity with official Ministry of Health guidelines. Finally, the fourth pertained to awareness of the decision-making process for individual appeals at the Ministry level.

**1. Engagement with Individual Appeals in Daily Work**

All QMs were familiar with the process and emphasized that submitting individual appeals was a routine and integral part of their daily work. A QM with more than a decade of experience at a large central medical center explained that there is always a “gray area” of cases within the individual appeals track. He noted that this gray area has persisted over time: sometimes a case may not fully meet the formal requirements of the quality indicator, yet it “remains clinically relevant or reflects a unique situation outside the scope of the indicator”.

Moreover, all QMs agreed that the individual appeals track is embedded in their routine responsibilities. A QM from a small peripheral institution, with seven years of experience, explained that submitting individual appeals had become almost automatic as she gained experience: “It’s ongoing, it happens with every report, every quarter. In cases that don’t meet the quality indicator criteria, if I see it is appropriate to submit an individual appeal, I do so.”

**2. Origins of Knowledge: Onboarding and On-the-Job Learning**

The QMs reported that they primarily learned about the individual appeals track through handovers from their predecessors, which varied considerably in length across institutions: from two weeks to as long as a year. Some QMs noted that during their handover period, they had no opportunity to manage individual appeal cases at all.

A QM with two years of experience explained that the individual appeals track was entirely omitted from her onboarding process. She admitted that “no one had explained it” and that she only became aware of the track later, through informal mentions by “more experienced colleagues”. According to her, the first formal introduction occurred during an inspection by a Ministry of Health supervisor. She added that her first actual use of the track occurred only after she had already “delved deeper into the role” and encountered numerous challenges in practice.

An experienced QM from a small medical center shared that she had to learn the track of individual appeals “on her own, through trial and error, while carrying out her routine work”. As a result, she believed that some justified appeals were never submitted, while others may have been submitted despite lacking sufficient clinical justification. She reflected:

*“It’s possible that at the beginning I missed some cases [of individual appeals] that should probably have been accepted. If I had the experience I have now*,* I might have submitted more cases for individual appeal. But that didn’t happen because of my lack of experience*,* lack of training*,* lack of guidance*,* the incomplete handover. In fact*,* I learned almost this entire role on my own.”*

This sentiment was also reflected in the voices of experienced QMs who noted that as they gained more seniority and experience, their individual appeal submission rates increased, as did the rate of appeals accepted by the Ministry of Health. One QM noted that “at first” it was difficult to understand the submission process, but later she “understood” and reached a point where “almost all the appeals I submit are accepted,” contrasting this with the beginning, when she admitted, “I really didn’t know.”

**3. Familiarity with Official Ministry Guidelines**

Interviews with QMs revealed that familiarity with the official guidelines issued by the Ministry of Health was generally limited. Only one QM was well acquainted with the published guidelines. Three QMs stated that no guidelines existed, while the remaining six acknowledged their existence but struggled to recall or describe their content. A QM with about five years of experience at a peripheral medical center, shared that she recalled a guideline being issued, including an example, but admitted: *“I honestly didn’t take it or print it out for myself*,* but I remember there was some kind of Excel file.”* Another QM with nearly three years of experience remarked: *“I never received any training on the topic [of individual appeals]. I don’t have any document that defines how to do it.”*

**4. Awareness of Ministry-Level Decision-Making**

Discussions about the individual appeals track revealed that the procedure is hierarchical, starting with an official request from the QM to the Ministry of Health and ending with a decision at the Ministry. However, the process at the Ministry level remained unclear to most interviewees: only two of ten QMs could describe it. The majority struggled to identify who makes the final decision on submitted appeals or which criteria are applied. For instance, a junior QM explained that, “to the best of their understanding, decisions were made by a supervising nurse”, although they admitted “not knowing the process in depth or being familiar with any formal work protocol”. A QM with nearly a decade of experience, echoed this uncertainty: *“I don’t know who reviews [the individual appeals] or from whom I receive the answer about them.”*

### Interactions among stakeholders

Interactions related to individual appeals occur across multiple levels and involve diverse stakeholders. Interviews revealed that QMs engage with clinicians, supervising nurses, the Ministry of Health, and, to a lesser extent, colleagues from other institutions to navigate the appeals process.

1. **Institution-level interactions**

All QMs reported that when clinical decisions are unclear, they consult with physicians and nurses, often drafting individual appeals collaboratively. A QM with ten years of experience explained that she reviews each case and submits an appeal when she deems it justified. When uncertain, she involves the physician, and more recently, she has worked alongside the physician to “review cases separately before reaching a final conclusion, creating a double-check process” that allows both perspectives to be considered.

Six QMs noted that before submitting an individual appeal, they often consult with the Ministry of Health’s supervisor nurse, whom they described as a “key resource” in clarifying cases and deciding whether an appeal should be submitted. All emphasized that communication with these nurses is “very good, close, and highly supportive.” A veteran QM from a large central hospital described her collaboration as “very fruitful and enriching,” explaining that she routinely reaches out to the supervisor nurse to “clarify issues” and receive guidance before finalizing an appeal.

2. **Ministry-level Interactions**

Regarding the interactions with the Ministry of Health, QMs’ responses were dual in nature. Six QMs emphasized that the individual appeals track makes them feel “heard” and provides an opportunity to express themselves and reflect the realities of frontline work to the Ministry. They also noted that the process reflects the Ministry’s flexibility and responsiveness to field-level concerns. At the same time, it serves as a feedback channel from the Ministry to the clinical field. A QM from the small hospital say: “And I know I’ll receive feedback on the Ministry of Health’s decision.”

On the other hand, four QMs shared their perspective, which was contrary to this understanding. In their opinion, the Ministry neither likes nor encourages the submission of individual appeals. Moreover, they feel the Ministry lacks awareness of what’s happening in practice and views each submitted appeal as an attempt to manipulate the system: *“Excessive bureaucracy. It’s like they don’t really believe me. They over-analyze every case… and I feel like the other side thinks I’m trying to trick them.”*

The QMs repeatedly stressed the importance of fostering “open dialogue and collaborative work with decision-makers” regarding the individual appeals track. These two needs recurred throughout the interviews and were reflected in proposals to enable QMs to receive personal feedback from the Ministry when an appeal is denied. Moreover, QMs would like to facilitate open dialogue with decision-makers on individual appeals through various channels such as professional forums, team meetings, and conferences. As one experienced QM from a large hospital explained, meaningful progress requires “a real discussion with the Ministry of Health and decision-makers, not just emails”, involving both the quality management and clinical teams alongside Ministry representatives.

In discussing the development of indicator definitions and standards for submitting individual appeals, a QM with three years of experience highlighted the need to “leverage the expertise of hospital quality staff” by strengthening collaboration between hospitals and the Ministry of Health. She suggested arranging joint meetings on both appeals and submission standards, noting that “the process isn’t quite clear.” According to her, involving hospitals in drafting these standards would create “a joint, evidence-based effort” grounded in real cases and current appeal data.

3. **Between- institution interactions**

All QMs stated that they were unaware of how the individual appeals track functions in other institutions. While some reported no contact with QMs from other hospitals and others maintained occasional communication, the topic of individual appeals “rarely comes up.” Although all participate in a national hospital quality forum on WhatsApp, they noted that even this platform is not used for discussing individual appeals.

While most QMs admitted they were not aware of practices in other institutions, one manager with three years of experience elaborated that there is “no real discussion” about individual appeals between hospitals, describing it as “a pretty closed world.” She added that QMs are “not necessarily encouraged to submit appeals,” noting that clearer guidance on “which cases can be appealed” might be beneficial and even “somewhat requested.”

The QMs also emphasized the need and desire for more open communication between institutions, highlighting that sharing experiences and practices could “improve understanding and management of individual appeals”.

### Expectation for professional acknowledge

The third theme that emerged from the interviews concerned QMs’ expectation of professional acknowledgment from the Ministry of Health. Beyond procedural or technical aspects, they viewed the individual appeals track as an important channel for professional validation and support, reflected across three subthemes.

1. **Expectations for Clinical Teams Professional Acknowledgment**

QMs emphasized that their role requires a delicate and ongoing balance between advocating for the needs and realities of clinical teams within their institution and meeting the standards, expectations, and regulatory requirements set by the Ministry of Health. As one experienced QM from a major central hospital described:

I’m actually in this position of… being a representative of the institution to the Ministry of Health, and at the same time, a representative of the Ministry of Health to the clinical staff… and that really places me in a position of dual representation.

Consistent with this view, QMs described the individual appeals track as a key “link between the clinical field and the Ministry of Health.” They emphasized that submitting appeals is “not just a technical task” but also a means to “reflect the hard work of clinical teams” and ensure their efforts are recognized nationally. As one QM explained, without the appeals process “you don’t get the real picture” of care quality, since some cases “don’t reflect the effort invested” and may unfairly lower performance rates.

2. **Expectation for QM Professional Acknowledgment**

QMs consistently emphasized the critical importance of “flexibility, empathy, and understanding from the Ministry of Health” in the context of the individual appeals track. They highlighted that beyond procedural compliance and data reporting, the process is deeply intertwined with the realities of frontline clinical work and the substantial efforts invested by management teams. QMs noted that acknowledgment of these efforts is essential for validating their professional contributions. One five years seniority QMs from a small hospital say: *“I think you*,* as managers in the Ministry of Health… need to understand what’s happening in the field… how much effort we*,* the management teams*,* invest just to achieve 100% performance in quality.”*

QMs revealed that individual appeals track is not merely as a technical or administrative exercise. Instead, it functions as a mechanism for professional dialogue and validation, enabling QMs to communicate the realities of patient care and operational challenges to the Ministry. Several QMs further highlighted that engagement in the individual appeals track conveys a sense of “being listened to and recognized”, which contributes to professional satisfaction and a stronger sense of ownership over quality outcomes: *“The process makes us feel heard and provides an opportunity to express ourselves and reflect the realities of frontline work.”*

3. **Acknowledgment through Acceptance of the Individual Appeal Request**

The expectation of professional acknowledgment was reflected in QMs’ reactions to the acceptance or rejection of individual appeals they submitted. When appeals were rejected, QMs expressed “frustration” and “a sense of being undervalued”, which they interpreted as a “lack of recognition” for their professional contributions. The senior QM from large hospital describes his reaction to rejection of individual appeal:

Bad. It [rejection] makes me feel bad. It makes me feel like I’m just doing meaningless coding work - not even clinical. And I feel like the Ministry of Health just wants to check boxes instead of doing real, in-depth review.

Conversely, acceptance of appeals was described as providing a sense of “closure of the circle, satisfaction, and professional validation.” A QM with three years of seniority explained that it brought “full satisfaction… it gives me peace, like a good feeling inside… Like, when the report file goes out, with the individual appeal included, I can relax - I feel I’ve completed that report.”

These contrasting experiences highlight that the individual appeals track carries not only procedural significance but also symbolic weight, serving as a mechanism through which QMs feel acknowledged and professionally validated by the Ministry of Health.

## Discussion

This study explored the perspectives of hospital QMs regarding the individual appeals track, highlighting both the challenges and the opportunities for strengthening this mechanism within the national quality indicators program. Using a phenomenological approach, the findings revealed gaps in knowledge, communication, and expectations for professional acknowledge, which have implications for national quality management.

### Knowledge gap

The findings from this study highlight a substantial knowledge gap among QMs in managing individual appeals track. While all QMs were familiar with the mechanism itself, many reported limited familiarity with Ministry of Health guidelines, often relying on personal judgment and experiential knowledge, which frequently outweighed formal guidance. QMs described learning primarily through handovers, trial-and-error, and on-the-job experience rather than structured training or standardized procedures.

These findings are consistent with prior studies indicating that many organizations often lack a robust onboarding process and ongoing training, both broadly [31] and within quality management roles. [32, 33] A recurring theme in the interviews was the reliance on tacit knowledge and “learning by doing” both during onboarding and throughout subsequent professional practice. While this hands-on approach to acquiring professional knowledge can enhance effectiveness, [34], [35] relying exclusively on experiential learning without integrating it into a structured framework may result in inconsistent practices [36] and varying levels of professional competence. [37].

Therefore, it is crucial that a structured mentorship program complement the onboarding process. In the current study, some institutions had such initiatives in place, while others did not. Evidence suggests that structured mentorship is highly effective in supporting overall integration during onboarding [31, 38]. Limited awareness of official Ministry guidelines further underscores the knowledge gap, highlighting a critical challenge in ensuring standardized and effective practice across institutions. This discrepancy is not unique to this context. Similar patterns have been documented in other healthcare systems, where clinical guidelines are often underutilized [39] unless accompanied by structured training. [40].

Notably, despite the publication of Ministry of Health guidelines in 2021, only one QM in this study reported being well acquainted with them. This suggests that the challenge lies not only in the existence of guidelines but also in their effective dissemination and integration into routine professional practice. Variability in onboarding processes, reliance on informal handovers, and the absence of structured training on the individual appeals track may contribute to this gap. From a policy perspective, these findings highlight the need for more proactive implementation strategies, including integrating the guidelines into formal onboarding programs and periodic national training sessions for QMs.

Addressing this knowledge gap requires the integration of a standardized, structured onboarding process combined with practical mentorship for all new QMs. This should be followed by continuous professional development and scheduled knowledge refreshers at predefined intervals for veteran QMs. This approach will ensure that all new QMs receive a uniform foundation of knowledge, while experienced QMs benefit from ongoing updates and systematic refreshers of existing knowledge and procedural guidelines. It also ensures that QMs not only receive experiential training but also have professional support and are fully informed and up to date regarding professional knowledge, official policies, and standards.

### Communication gap

The findings of this study reveal that, from the perspective of QMs, communication with Ministry nurse supervisors and with clinical partners within hospitals was described as close, supportive, and highly valuable for managing the individual appeals track. However, QMs also reported limited clarity regarding decisions at higher organizational levels, and a lack of personal feedback from the Ministry, along with weak communication with peer institutions. Thus, the gap was partial: while effective collaboration exists at the local and supervisory levels, disconnection is most evident with the higher tiers of the Ministry of Health and across institutions.

Such gap is concerning, as prior research has consistently demonstrated the critical role of communication for organizational functioning. Bartels et al. (2010) [41] demonstrated that vertical communication predicts organizational identification, while horizontal communication predicts professional identification that are both essential for balancing institutional and professional responsibilities. Extending this, Zhang et al. (2020) [42] showed that vertical and horizontal communication strengthen organizational commitment via employees’ perceptions of job attractiveness and service performance, suggesting that more open dialogue could enhance engagement and motivation. In addition, Gbarale et al. (2020) [43] found that vertical communication contributes significantly to employee effectiveness and efficiency, with organizational culture moderating this relationship, highlighting that without a supportive culture, communication structures may fail to achieve their potential.

Taken together, the literature suggests that effective vertical and horizontal communication is a key driver of identification, commitment, and performance. The communication gaps observed in this study therefore represent a missed opportunity: clearer feedback mechanisms and stronger inter-institutional dialogue could support QMs’ professional development and improve but also system-wide quality outcomes. Beyond these communication challenges, the findings point to structural ambiguity in how individual appeals are reviewed at the Ministry of Health. Many QMs reported uncertainty about who determines the outcome of appeals and the considerations guiding these decisions. Establishing a transparent operational framework (by defining review stages, roles, and evaluation principles) could enhance consistency and predictability while reducing the frustration and perceptions of arbitrariness expressed by interviewees. Clarifying these procedures may strengthen trust between hospitals and the Ministry and improve the functioning of the appeals mechanism.

### Meaningful recognition gap

The findings reveal that QMs have a pronounced need for recognition from the Ministry of Health for their efforts. In practice, however, they receive little to no feedback, leaving them feeling unheard, frustrated, professionally insignificant, and demoralized. These emotions are particularly pronounced when appeals are rejected, even if clinically justified. QMs often perceive such rejections as a dismissal of their expertise and effort, underscoring a lack of appreciation for the nuanced work required to uphold quality standards. This longing for acknowledgment reflects the gap in meaningful recognition not only from immediate supervisors but also from higher levels within the Ministry.

According to the concept analysis by Eddy et al. (2021) [44], meaningful recognition occurs when individual values and goals align with organizational values and goals, creating a synergy. The literature highlights that meaningful recognition extends beyond generic praise or token acknowledgment, encompassing deliberate actions that validate employees’ contributions and professional expertise. Studies indicate that staff whom experience meaningful recognition report higher levels of engagement, job satisfaction, and organizational commitment, which in turn enhance overall performance and foster a healthier work environment. [45–47].

Based on Eddy et al. (2021) [44], meaningful recognition can be expressed through both organizational and personal actions. At the organizational level, this includes establishing structured recognition programs that reflect and reinforce the organization’s mission, publicly acknowledging employees’ contributions in meetings or communications, and providing formal mechanisms for feedback and professional development. At the personal level, meaningful recognition involves offering individualized feedback that highlights employees’ expertise, effort, and accomplishments, expressing gratitude for their work, and supporting them in documenting and communicating their contributions.

Drawing on the recommendations of Joseph et al. (2023) [48] for cultivating a culture of meaningful recognition, structured strategies should be implemented to validate and celebrate the contributions of QMs. Key actions include establishing regular, individualized forums for communication between QMs and Ministry staff; formally acknowledging the specialized expertise, clinical judgment, and ongoing accountability inherent in their roles; and tracking the outcomes of their appeals to reflect both hospital-level and organizational-level impact. QMs’ efforts should be visibly recognized in Ministry-wide communications, while sustainable recognition initiatives, including formal feedback, equitable acknowledgment, and expressions of gratitude, should be embedded at all organizational levels.

### Exception mechanisms in practice

This study highlights challenges in implementing exception mechanisms within large-scale quality measurement systems. While standardized indicators enable benchmarking and accountability, they may not fully capture the clinical context of individual cases. [49] Appeals provide a formal mechanism for documenting situations where adherence to an indicator is not clinically appropriate,^13^ helping reconcile the tension between standardized measurement and professional judgment in complex cases, as noted by QMs in this study.

The practical importance of appeals also depends on indicator characteristics. For indicators with small denominators or rare events, including or excluding a few cases can meaningfully affect performance. For high-volume indicators, the statistical impact of individual appeals is limited. [50, 51] QMs reported that while appeals rarely alter high-volume indicators, they remain important for addressing atypical cases and ensuring reported results accurately reflect clinical realities. In this way, appeals contribute both to methodological refinement and to maintaining clinicians’ trust in the fairness and credibility of the quality system.

Submitting quality indicator data entails significant administrative effort, including documentation and justification for both general and case-specific appeals. [52] This workload raises questions about efficiency and scalability. Even when appeals minimally affect overall performance, a structured review process contributes to the legitimacy and acceptance of national quality programs. By providing a formal pathway for clinicians and QMs to explain exceptional cases, appeals sustain engagement with performance measurement systems and mitigate concerns that indicators are applied too rigidly or detached from clinical realities.

In summary, this study offers unique insights into hospital QMs’ perspectives on the individual appeals process, highlighting both challenges and opportunities for strengthening this mechanism within the national quality indicators program. The findings reveal gaps in knowledge, communication, and meaningful recognition, reflecting broader aspects of organizational culture. A key strength of this study is that, to the best of our knowledge, it is the first to capture the lived experiences of QMs in this context. Moreover, the study provides an evidence-based foundation for practical, national-level recommendations and emphasizes the importance of a multifaceted approach to fostering a culture of cooperation and transparency.

### Limitations

This study has several limitations that should be considered when interpreting the findings. First, the research relied solely on the perspectives of hospital QMs, which may not fully capture the experiences or viewpoints of other stakeholders. Second, the phenomenological approach, while providing in-depth insights into QMs’ lived experiences, is inherently subjective and may be influenced by participants’ recall bias or personal interpretations of the individual appeals track. Third, while theoretical saturation was achieved, the sample size was modest, and representation across different hospital types was limited, so the findings should be interpreted with some caution when generalizing to all hospital contexts. Finally, cultural and organizational factors specific to the local context may influence the applicability of these findings to other healthcare systems.

## Conclusion

This study highlights significant gaps in knowledge, communication, and meaningful recognition among hospital QMs involved in the individual appeals track. Addressing these challenges requires a multifaceted strategy: implementing structured onboarding and mentorship programs, fostering transparent vertical and horizontal communication, and establishing formal mechanisms for meaningful recognition.

In addition, the findings highlight the potential value of establishing structured national learning and training frameworks for hospital QMs. Formal training programs and periodic joint meetings involving QMs together with their counterparts at the Ministry of Health could help address the gaps identified in this study. Beyond strengthening procedural knowledge, regular meetings could also foster a stronger professional community among QMs and promote a shared understanding of the rules governing the individual appeals process.

Strengthening these areas can enhance QMs’ competence, motivation, and engagement, while promoting a culture of cooperation and transparency across organizational levels. Embedding these cultural values into organizational practices can build trust, reinforce professional commitment, and support shared accountability, ultimately fostering more consistent, effective, and sustainable quality management across hospitals.

## Data Availability

The datasets used and/or analyzed during the current study are available from the corresponding author on reasonable request.
